# Report of the Diseases of Edinburgh for November, 1807
*This Report, by some unavoidable accident, was not received by the Editors until the 23d of the present month, (February.)


**Published:** 1808-03

**Authors:** John Roberton

**Affiliations:** Surgeon


### *Report of the Diseases of Edinburgh for November,* 1807.*

By Mr. John Roberton, Surgeon.

The weather, for the first ten or twelve days of this month, was so uncommonly variable, that an almost complete change in its nature occurred every few hours, yet it

---

* This Report, by some unavoidable accident, was not received by the Editors until the 23d of the present month, (February.)

never was, during this time, intemperate. That immense range of mountains, about north-west from this city, and distinctly seen from several parts of it, was, early in the month, covered with snow. This is very frequently a warning to us of what we are to expect in that way ourselves. The thermometer in Edinburgh and its vicinity stood, for the most part, about 40, and the barometer was, as might be expected, extremely unsteady. About the middle of the month, the weather was more severe, and we had some showers of hail and rain, and, once or twice, slight falls of snow. Near the end of it, the weather became extremely tempestuous, sometimes attended with a very unusual degree of cold, and the snow fell in considerable quantity. For a day or two about this time, the thermometer was uncommonly low; standing, in the immediate neighbourhood of this city, at 16, and within the city at 20. Before the termination of the month, a greater quantity of snow had fallen than has, for many years, been known in this country at so early a period of the season.

Inflammatory Diseases, both among adults and children, have been very prevalent, and, in many instances, they have proved suddenly fatal. These complaints had even made their appearance before the termination of last month, and, in several instances, I found that those patients who had recently suffered from the febrile affections mentioned in my last Report, were very liable to them in the form of Enteritis. The inflammatory diathesis that prevailed did not seem to be confined to people of a robust habit of body, as it frequently affected those persons who had been much reduced by other diseases; and I have been informed, that some women who had been delivered immediately, and many who had been delivered several weeks before, were seized with inflammation of the uterus.

The mode in which these diseases usually attack people was this. Infants and children were affected with Enteritis, and people advanced in life with Pneumonia. The Pneumonia, as it at present prevails, often for a few hours at first, in its symptoms resembles Catarrh, but the transition of these symptoms to the most acute Pneumonia is indeed very sudden, and, if the most active treatment is not immediately employed, very serious consequences are to be expected. I have known some patients die on the 2nd, and some on the 3rd day of the disease. The abstraction of very large quantities of blood is instantly to be had recourse to, with the application of large blisters to the chest, and and the free use of purgatives. Even in the course of thirty hours, I have found it absolutely necessary to take from one patient, at three different times, upwards of seventy ounces of blood; which exhibited the strongest marks of inflammatory diathesis. The first bleeding not so much as the second, and the third less than either. This very active treatment I have almost always found necessary to remove completely most of the cases of Pneumonia I have lately seen.

Early in the month the febrile complaints mentioned in last Report, unattended by inflammatory symptoms, seemed to gain ground, and patients, after suffering from that exhaustion inseparable from every description of fever, were, in many instances, seized with symptoms of inflammation, frequently of considerable violence. In that state of debility, blood-letting, which, but for this circumstance, seemed indespensibly necessary, could not be employed. Blistering, with gentle doses of saline purgatives, was, in such cases, the only remedy from which benefit was obtained.

Chin Cough still continued very prevalent among children, but its fatality does not seem to be so great as we have had occasion to observe it, even where the disease has been much less frequent.

Catarrh has likewise been of so frequent occurrence, that whole families have been affected with it. In some it has been combined with Cynanche Tonsillaris, attended with considerable feverishness, and with a dry hard cough, particularly after it had continued for some days. The catarrhal symptoms, when unattended by fever, have, in general, been removed by purgatives, diaphoretics, and strict attention to the removal of every species of dampness. The Cynanche was somewhat more obstinate, not yielding to the exhibition of saline purgatives and gargles of different kinds, till, in addition to these, the repeated application of blisters to the throat, extending from one angle of the jaw to the other, was employed. The symptoms then gradually abated, and, when this practice was early had recourse to, I have not met with any case that has terminated by suppuration. I may remark, that in one very obstinate case, which had been rendered so by the patient having neglected to inform me of his indisposition, till after the inflammation had continued nearly a fortnight, his throat became one mass of ulceration, and even the whole upper surface of his tongue, within a little of the point, was deeply ulcerated. These appearances

 alarmed alarmed him very much, as he had recently suffered from a venereal complaint, and suspected that the present appearances were, in consequence of the virus still lodging in his system. The symptoms were, however, subdued by blisters applied to his throat, by gargles, and the use of purgative medicines.

It may not be improper, in this place, to take notice of that species of Catarrh which frequently happens to people advanced in life, who, from frequent repetition of catarrhal affections, become habitually affected with a sort of Chronic Cough, often continuing for many years, and proving extremely distressing. Its attacks are most common in the morning, and the ill fated patient, otherwise in very good health, is seized with fits of coughing for hours together, till free expectoration is produced and relief obtained. Next morning, however, the same distressing symptoms again seize the enfeebled patient, and thus, the little strength he may possess to support him through the fatigues of the day, is nearly exhausted. This is a disease very common in our northern climate. It seems to arise from an unusual quantity of mucus secreted in the bronchiæ, and, perhaps, in the lungs, which, by impeding respiration, or mechanically irritating these parts, produces the cough. In such cases, I have employed the following pills with very decided success.

℞. Gum. myrrh ʒj. Gum. ammon. ʒß. P. R. scill. gr. x. opii gr. vi. Syr. simp. q. s. f. massa in pil. gr. v. dividenda.—Sig. Two to be taken morning and evening.

After continuing their use for a considerable time, which is often necessary in these Chronic Coughs, I have increased the dose to six or even more in the course of 24 hours.

When the inflammatory symptoms of Catarrh have been subdued, and the cough alone has continued, I have repeatedly found these pills to possess a surprising effect in promoting expectoration and removing the disease. In some severe cases of long standing, where they have become chronic, an almost daily use of these pills is found to be necessary. I know that the inhalation of the steams of vinegar and warm water at bed time, is very useful; so also is the internal use of oily and saccharine matters, by besmearing the fauces, and thus giving momentary relief; but the practice I have now recommended seems to me preferable.

Partial Inflammation about the glottis, which some people, during the winter months, are subject to, even from the slightest causes, produce a cough which, to the partial partial observer, may be mistaken for the Chronic Cough now mentioned; but, in this, the same practice is not to be depended. Relief is only to be expected from the application of blisters to the throat. We all know that by the proper discrimination of complaints from one another, our professional knowledge is to be estimated. It, perhaps, may appear unnecessary to relate a case in proof of this; but as the following one was, in many respects curious, I shall give a brief account of it. One of my reasons for introducing it here, is on account of its having been long treated as a Catarrh, and afterward as consumption, but it happened to be neither of them. I shall relate it precisely, as it was given to me. The patient was a blacksmith, who, while dining, swallowed a small piece of bone. It, at the time, occasioned a good deal of coughing, which was followed by considerable inflammation of the throat. This was subdued; but still the cough continued to trouble him, and he was purged and blistered, but derived no benefit from them. He complained of no particular pain or difficulty of swallowing, yet he became gradually debilitated and feverish, to such a degree, that he was at length unable to attend his business. All the different medicines that, perhaps, had ever been prescribed for the removal of a cough, were given him, but he obtained no relief from them. About the seventh month of his disease, he became pale, emaciated, and evidently hectic, and he was almost constantly troubled with shooting pains in his chest: night sweats also contributed to debilitate him very much. His complaint was now thought to be pulmonary consumption, and underwent such treatment as was thought proper for that disease; but still he reaped no relief, became tired of drugs, and would have no more of them. About a month afterward, a violent fit of coughing seized him, which his friends thought would terminate his existence, and during it, he, in the act of retching, spit up a piece of bone, very much decayed, which was evidently the remaining portion of what he had swallowed eight months before. His complaints soon after entirely left him, and he is now working at his trade as healthy as ever he was in his life.

In many of these people who are affected by Chronic Catarrh, and have been much exhausted by old age or by a repetition of enfeebling complaints, the power of expectoration ceases, while the bronchial secretion goes on; the patient becomes drowsy; the skin cold; the pulse small and fluttering; the face tumefied and discoloured;

 the the lips livid; the breathing short and difficult, till, at last, the bronchiæ are so replete with mucus, that the admission of a sufficient quantity of air to support life becomes impossible, and he is suffocated.

Many diseases, apparently different in their nature, are produced by the same cause. It is owing to some peculiarity of the constitution of different individuals, or inequality of the parts with regard to their powers of resisting morbid action, that the same application of temperature, that, in one person, would excite diarrhœa, cynanche tonsillaris, pneumonia, or rheumatism, will induce catarrh in others. The cause of this peculiarity is difficult to be accounted for.

*4, St. James's Street.*

---

